# 3D Point Cloud Verification and Scatter Removal Using a Dual Camera/Projector Setup

**DOI:** 10.3390/s25237250

**Published:** 2025-11-27

**Authors:** Jens T. Thielemann, Jostein Thorstensen

**Affiliations:** SINTEF AS, 0373 Oslo, Norway

**Keywords:** subsea imaging, 3D imaging, scattering media

## Abstract

**Highlights:**

**What are the main findings?**

- Scattering media like water creates false 3D data points when using active illumination. This effect complicates subsequent machine vision tasks such as boundary detection, size estimation, and object segmentation.
- Our method can remove up to 99% of such false 3D points, making it an enabling methodology for robust and trustworthy 3D imaging in turbid conditions.
- The algorithm is O(1), meaning that it can be made suitable for embedded platforms and real-time systems.

**What are the implications of the main findings?**

- The method enables high-quality, turbidity-independent 3D imaging for underwater robotics, inspection, and autonomous operations, even in challenging environments.
- The reliable removal of scatter-induced artifacts allows accurate object boundary detection, critical for tasks like manipulation, navigation, and monitoring.

**Abstract:**

This work investigates the properties of 3D imaging in turbid water in context of being able to precisely remove false 3D measurements that are a result of scattering. Such false measurements make object boundaries highly uncertain and limit the usefulness of 3D data. The work is motivated by a need for high-quality 3D image data for underwater robotics, also in turbid waters, where false 3D data can make 3D point cloud algorithms complex and unreliable. Basing ourselves on triangulation-based 3D imaging in turbid water, we show through first-principles analysis how a combination of certain camera/projector geometries and projected patterns allows for the reliable discrimination between true and false 3D measurements. We present an efficient ($O\left(1\right)$) algorithm capable of rapidly performing this discrimination. Through experiments, we show that we can provide a 50–100× better false positive rate than single-camera algorithms across relevant turbidities, whilst maintaining an almost zero false negative rate. This combination of camera hardware, projected patterns, and algorithms gives a significant robustness improvement of subsea 3D imaging systems, enabling robust and trustworthy 3D imaging.

## 1. Introduction

Underwater robotics is a market in rapid growth, driven by applications in oil, gas, and mineral exploration, telecommunications, renewable energy infrastructure monitoring, and sea life and environmental monitoring [1]. For the land-based industry, optical 3D imaging is a key technology enabling precise observation, measurement, and object identification as well as robotic interaction. Similarly, we expect high-performing 3D cameras to also be required for underwater robotics. By high-performing, we mean 3D measurements with millimeter or sub-millimeter XYZ resolution, which is only achieved with structured light imaging, laser line scanning, or other multi-frame triangulation-based methods. For robust operation in real-life environments, 3D cameras will need to be able to operate in turbid waters.

When reviewing the literature on optical underwater 3D imaging, we find many studies on passive 3D imaging techniques such as Structure from Motion (SfM) and Photogrammetry (reviewed comprehensively in [2]), and more advanced methods like integral imaging [3,4,5], Neural Radiance Fields [6] and transformer/deep-learning-based methods [7,8]. While these methods are good for large-scale structures, they do not provide the 3D data quality required for robotic interaction, as they require object texture to provide the detailed 3D information necessary for precise interaction. Furthermore, passive techniques generally assume the availability of ambient illumination, which limits their usefulness in the deep sea. Time of Flight (TOF) methods have also been developed for underwater applications, but generally provide depth accuracy in the cm range (reviewed in [9]), or perform single-photon measurements for sub-millimeter depth resolution, however, with the cost of exceedingly long exposure times (30 min) [10].

For subsea imaging, scattering is a core limiting factor for 3D imaging. The scattering can be divided into backscatter (light reflecting off particles in the water without interacting with the objects in the scene) and forward scatter (light deflected on its way from the projector to the object or from the object back to the camera). The scattering will make the 3D data incorrect, as it introduces seemingly good 3D data when actually observing a void, and it blocks out darker objects from observations. This limits the usefulness of 3D cameras for advanced interactions.

For references using structured light imaging, several papers report results only in clear water [11,12,13]. Few authors report on turbid water, with some authors choosing milk as a scatterer [14,15], and others use clay [16,17,18], presumably more representative of real waters. NTU (Nephelometric Turbidity Units, as defined by ISO 7027-1:2016) is often used as a standard for measuring water turbidity through measurement of scattered light intensity at a 90° scattering angle. This means that NTU is only representative for water scattering if the angular scatter distribution and light absorption are in agreement with what is found in real waters.

Previous methods to reduce the impact of scattering include range gating [19], using a prior to subtract it in postprocessing [17], use of well-selected patterns [20], or the use of image sharpening [21] methods. All these methods share the ability to reduce the effect of scattering, but they are not able to provide any verification of whether the scattering was successfully managed. Furthermore, they are not able to reliably determine whether a received signal is due to an object being present in the scene or merely being an artifact of the scattering. Many such methods need to be closely tuned (e.g., algorithmic parameters) to the expected turbidity, scene geometry, and reflectivity.

In this paper, we present a novel first-principles method that enables reliable discrimination between real signals and scattered signals. By using two cameras and a single projector as our imaging setup, we design an imaging geometry that allows for independent observation of the 3D scene, and that ensures that any errors introduced by scattering can be detected, measured, and removed on a first-principles basis whilst preserving real signals. We further provide a framework for classifying observations according to the level of trust that can be put into them. We measure the effectiveness of our method by considering it as a classifier, and report on its ability to discriminate true 3D measurements from false 3D measurements that are due to scattering.

**Figure 1:**
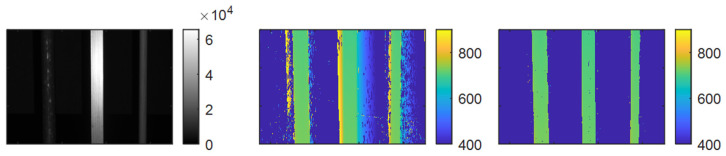
Illustration of the scattering problem. Left: Amplitude image of the scene, showing three poles immersed in somewhat turbid water (NTU 1.6). Middle: Corresponding 3D data from the scene. We see that due to scattering, false 3D data is reported outside the poles due to forward scattering in the scene. Right: Result after removing false data points due to scattering by using our method.

Our method enables the precise determination of object boundaries using depth data. Normally, when observing isolated objects (e.g., free-swimming fish or smaller mechanical structures), the distinction between object and background/void will be highly unclear, making object size estimation and localization highly uncertain. An example is given in Figure 1, where we see that the 3D data of a scene imaged through turbid water blurs out and makes the 3D data highly uncertain in terms of determining object boundaries. By contrast, our method successfully removes data points due to scattering whilst preserving the actual 3D objects fully. Our method does not rely on signal amplitude, meaning that dark objects can be successfully imaged whilst still removing scattered data points. By being able to determine which 3D data points can be trusted, object boundaries can be reliably used for size estimation and object localization. This can enable future applications within robotics, aquaculture, and autonomous operations.

The rest of the paper is organized as follows: Section 2 introduces underwater 3D imaging by triangulation and how scattering affects these kinds of measurements. Section 3 presents our camera setup and calibration process, and the principles and methods for scatter removal from 3D measurements. In Section 4, we report our qualitative and quantitative results applying our method, before concluding in Section 5.

## 2. Theoretical Background

### 2.1. Short Introduction to Underwater 3D Imaging by Triangulation

**Figure 2:**
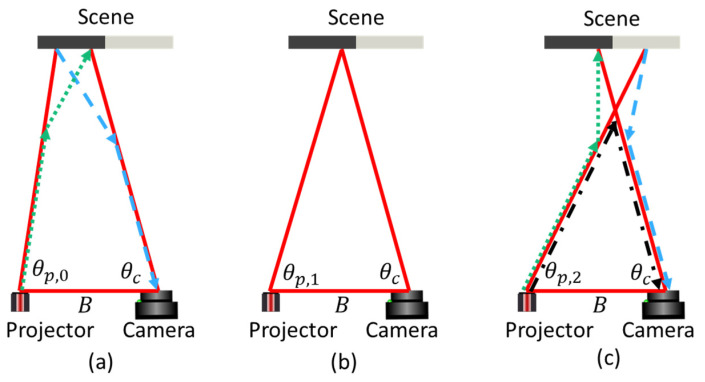
(**a**) t = 0: Forward-scattered signal (blue and green). (**b**) t = 1: Direct (ballistic) photon transport. (**c**) t = 2: Forward scatter (blue and green) and backscatter (black). Scattering occurs at particles or impurities distributed in the water volume.

3D measurements in the geometry shown in Figure 2 are obtained by triangulation from the observation at an angle $\theta_{c}$ of a projected pattern emitted at an angle $\theta_{p,1}$ at a baseline distance B through the relation.

$$D=\frac{B\;sin\left(\theta_{c}\right)}{\sin\left(\theta_{c}+\theta_{p,1}\right)}$$

In practice, one may select several strategies for ensuring a return signal in the pixel with an observation angle $\theta_{c}$. For now, we shall assume that we project a line perpendicular to the baseline, and that we vary the projection angle to span the entire field of view of the projector in $t=[1,t_{max}]$ discrete time steps $\theta_{p}\left(t\right)=-\theta_{p,max}+\frac{2\theta_{p,max}t}{t_{max}}$. For the simple case shown in Figure 2b, the camera observes, at $\theta_{c}$, a bright signal $I\left(t\right)$ at the time when $\theta_{p}(t)=\theta_{p,1}$.

However, for underwater imaging, scatter will also be present. Light, normally traveling in straight paths, will undergo a change in direction when interacting with scattering particles in the water. The most prominent (first-order) light transport mechanisms are shown schematically in Figure 2a,c. Higher-order light transport (i.e., more than one scatter event) will also be present, but for this discussion, we shall assume that they are less likely than the first-order transport. We keep the observation angle $\theta_{c}$ fixed and let the projected line scan from left to right.

At t=0 (Figure 2a), the projected light does not intersect with the ray observed at $\theta_{c}$. In air, the camera would observe no signal in this situation. However, underwater, forward scatter enables photon transport along the green and blue rays. At t=1 (Figure 2b), the direct ray from the projector reaches the scene point observed by the camera, and the direct light transport route is observed. At t=2, the projected light crosses the ray observed at $\theta_{c}$ in the water volume. Forward scatter (dotted, green and dashed, blue) is still present. Additionally, the light traveling from the projector towards the scene can undergo backscatter in the water volume at the point where the projected light and the camera ray intersect (dash-dotted black).

In this simplified discussion, we chose to project only one single line, which was shifted in time, in a pattern projection scheme often referred to as “laser line scanning”. This is the configuration used, e.g., by Narasimhan [14] and, as noted by Narasimhan, it has the advantage that the light transport paths discussed above will appear at different times in the recorded image sequence, thereby greatly simplifying the signal analysis. In practical 3D reconstruction, one will find the maximum of $I\left(t\right)$ observed at $\theta_{c}$, and assume that this is representative of the situation at t=1, where the triangle is closed, and triangulation can be performed to yield the correct distance to the scene. This assumption may or may not be true, as the signal from the different transport paths will depend both on the water scatter and attenuation properties, and on the scene reflectivity/geometry. A somewhat subtle point is that correct 3D reconstruction requires the situation described in Figure 2b, where the light paths are straight. This means that only the unscattered, “ballistic” photons contribute to the 3D measurement. The unscattered signal retains its spatial frequency, meaning that even in scattering media, the true signal peak remains sharp [20].

For 3D imaging, not only is “laser line scanning” possible, but sine wave projection is often used, where the entire scene (all $\theta_{p}$) is illuminated simultaneously, but with different local intensity [17,21], thereby forming a sine wave in the spatial domain. With such an illumination strategy, it becomes difficult or impossible to separate the different light transport paths, and the measured $\theta_{p}$ used for distance estimation may end up being a weighted average of the individual contributions. In a previous publication, we found that, while sine-wave patterns may be more light-efficient in clear waters, they do not uphold performance in more turbid water, where the projection of one or more lines yields superior performance [20].

### 2.2. Influence of Scatter on 3D Data Quality

In the previous section, we stated that we can, in many cases, retrieve the situation found in Figure 2b by projecting a line pattern and finding the maximum signal for a given pixel. We shall now discuss the most common situations where this approach will fail, and their influence on 3D data. We shall discuss three groups of situations:

1. Backscatter-dominated signal.
2. Forward-scatter-dominated signal.
3. 3D data outside of physical objects.

For a comprehensive review of the optical properties of water, please see, e.g., [22]. Three-dimensional data outside of physical objects is a particularly difficult and often overlooked problem when it comes to underwater 3D imaging, and it is important to understand the scattering process to understand why false 3D data is reported.

#### 2.2.1. Backscatter-Dominated Signal

**Figure 3:**
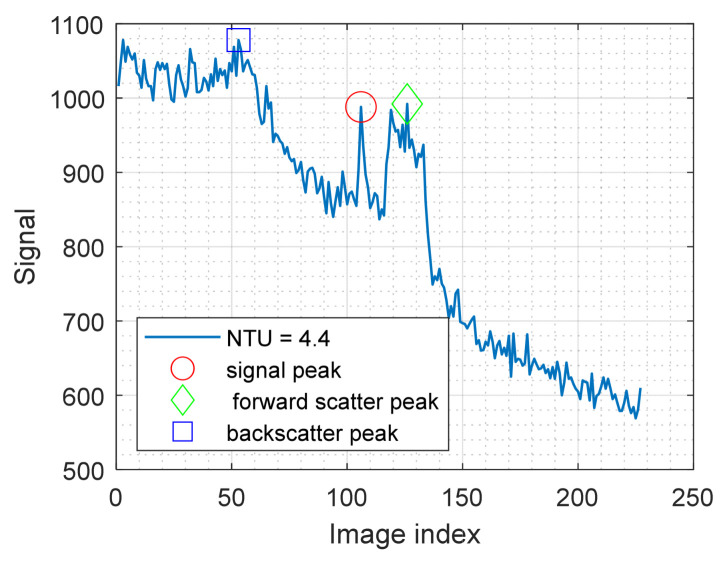
Example signal where both backscatter (blue square) and forward scatter (green diamond) are higher than the true signal peak (red circle).

Backscatter is light that is scattered by the water itself before the light hits an object, shown schematically with black arrows in Figure 2c. The scatter occurs throughout the water volume, and the angular distribution of the backscatter is relatively flat (ref Figure 10 in [22]), making backscatter a broad feature both spatially and along the time axis (with t as defined in Section 2.1). An example signal with significant backscatter is shown in Figure 3, where the maximum signal at the backscatter (blue square) is higher than the signal from the object (red circle). When choosing to project a thin line, it can be noted that the signal from the object itself is sharp, both spatially and in time. This behavior opens up the possibility of performing filtering (either in the spatial or the temporal domain). This approach has been attempted both with time-of-flight imaging and with structured light imaging [17,21], through the use of unsharp mask filtering or similar high-pass filters. It should be noted that while it is possible to significantly reduce the backscatter envelope through high-pass filtering, the photon shot noise (being white noise) will, of course, remain in the filtered signal. With typical signal levels, shot noise can be 10–100 times higher than the camera read noise, causing a similar increase in detection limit.

#### 2.2.2. Forward-Scatter-Dominated Signal

Forward scatter is light that is scattered in the forward direction through the water volume, as indicated in Figure 2a,c. Forward scatter can occur either as light travels towards the object or as the light returns towards the camera. An important feature of forward scatter is that the amount of forward scatter reaching the camera is dependent both on the scatter probability and angular scatter distribution in the forward direction, and on the reflectivity of the object at the point where the light hits. In Figure 2c, the green arrow indicates forward scattering occurring towards the object. When the light hits a dark part of the object, the resulting signal is low. The blue arrow, on the other hand, hits a bright part of the object, and the corresponding signal is stronger. If the camera observes the dark part of the object, the signal from the blue path can be stronger than the signal from the object, leading to erroneous 3D reconstruction.

While backscatter is slowly varying in space and time, forward scatter carries with it the spatial frequencies of the reflectivity in the scene, meaning that it can exhibit high-frequency variations in time (when scanning $\theta_{p}$). The angular scatter distribution is also highly peaked in the forward direction, meaning that the signal is strongest at small scatter angles (ref Figure 10 in [22]), making the forward-scatter high-frequency in the spatial direction as well.

Similar to high-pass filtering of the signal for backscatter removal, it will be possible to identify filtering strategies that are suitable to suppress forward scatter in certain scenes and at given turbidity levels. Examples can again be high-pass/band-pass filtering, peak prominence, or peak width metrics. Common to these filters is that they attempt to target the spatiotemporal signature of the true signal, while assuming that the signature of forward scatter does not carry similar components. As stated above, this may or may not be true, depending primarily on scene texture and geometry. Further, the performance of such filtering strategies declines with low signal-to-noise ratio signals.

#### 2.2.3. Three-Dimensional Data Outside of Physical Objects

When discussing forward- and backscatter, we have implicitly discussed it in the context of filtering out the “wrong” signal to obtain the “correct” signal. However, especially in subsea applications, it is interesting to observe what happens to our 3D data outside of physical objects, i.e., when there is no “correct” signal to be found. In the absence of scattering, no signals will return from these parts of the scene, and the signal will be noise only, which can be filtered by peak thresholding, median filtering in the z-domain, etc. However, in scattering media, both forward- and backscatter can contribute to the signal in these parts of the scene. As shown in Figure 3, these signals can easily be higher than signals that we are interested in detecting in other parts of the scene (i.e., dark or far away objects), resulting in the false 3D data shown in Figure 1. One will inevitably end up in a tradeoff between filtering out weak, true signals and keeping false data points from scattering. Additionally, median filtering will be inefficient, as the scatter will provide spatially consistent data points. Three-dimensional data points from scattering will form a halo around objects, making object avoidance, object segmentation, and pose estimation difficult or impossible, while the removal of weak, true signals presents similar problems due to missing information.

**Figure 4:**
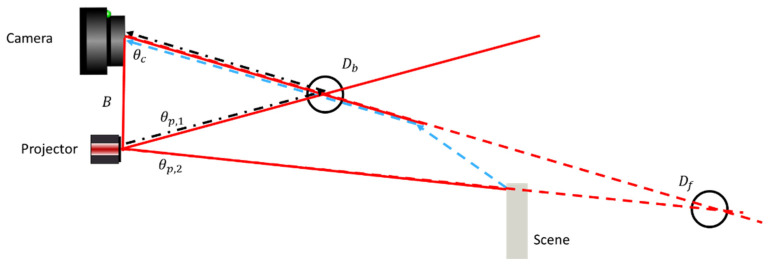
Three-dimensional data outside of an object. Backscatter (black, dash-dotted) forms a maximum signal when projecting at $\theta_{p,1}$, perceived as an object at distance $D_{b}$. Forward scatter (blue, dashed) forms a maximum signal when projecting at $\theta_{p,2}$, perceived as an object at distance $D_{f}$. The red-dashed lines indicate the viewing angles of the pixels of the camera/projector that observe/contribute to the false forward scatter object detection.

The situation is illustrated in Figure 4. Backscatter (black, dash-dotted) forms a maximum signal when projecting at $\theta_{p,1}$, and the projected line and the field of illumination intersect within the water volume. From triangulation with the observation line along $\theta_{c}$ and projection line along $\theta_{p,1}$, this maximum signal is perceived as an object at distance $D_{b}$. Forward scatter (blue, dashed) forms a maximum signal when projecting at $\theta_{p,2}$ and the projected line hits the object. This will be perceived as an object at distance $D_{f}$. Note that all camera pixels $\theta_{c}$ observing the outside of the object will observe a forward-scatter peak when projecting along $\theta_{p,2}$, resulting in many peak observations at $\theta_{p,2}$, all with different perceived $D_{f}$. Observation at the opposite edge of an object puts $D_{f}$ closer to the camera. This behavior is not limited to regions outside of an object. The same holds if observing a sufficiently dark part of an object, where either forward- or backscattered signals are stronger than the true signal from the object.

As a side note, if considering a Time-of-Flight (TOF) system, $D_{b}$ will also occur in the same position temporally, being observed as an object closer to the camera. However, as the blue dashed line is approximately the same length as the distance to the object, $D_{f}$ will, in a TOF system, appear as a skirt around the object at the distance of the object.

## 3. Materials and Methods

### 3.1. Camera Setup and Calibration

**Figure 5:**
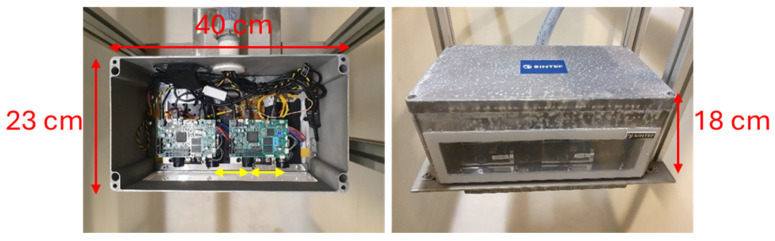
Experimental underwater 3D camera setup. Size: 40 × 23 × 18 cm (W × L × H). The two 75 mm baselines are marked with yellow arrows.

We have built the camera shown in Figure 5. It consists of two cameras, with a projector in the middle, each separated by a distance of 75 mm. The projector is a Texas Instruments LightCrafter4500 (Texas Instruments, Dallas, TX, USA), while the cameras are Basler ace U acA1440-220uc (Basler AG, Ahrensburg, Germany). The housing is watertight, allowing images to be captured underwater. A single-camera version of the setup was described and characterized in a previous publication [20], and was found to reconstruct 3D with a precision of 0.2 mm at 1 m distance, at turbidities up to NTU 4. As will be discussed in Section 3.2 and Section 4, the dual camera setup will, by design, retain this precision. Further details can be found in [20].

The premise of our method is that an observation in Camera 1 can be confirmed in Camera 2. This requires that both the cameras and the projector are calibrated both intrinsically (lens parameters) and extrinsically (relative position of components). Calibration must take into account the refraction occurring at the air/glass/water interface and was therefore performed in clear water. In our previous publication, the reconstructed distance was constant across turbidities within our measurement precision, a good indication that calibration can safely be assumed to be invariant with turbidity.

**Figure 6:**
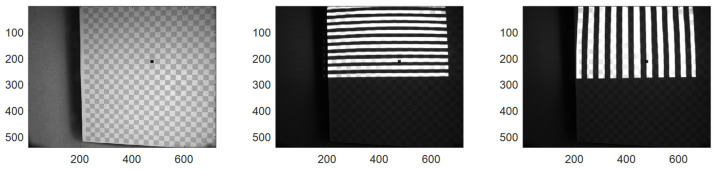
Example calibration images. **Left**: Camera image of a checkerboard with a black dot to ensure corner identification, using ambient room illumination. **Mid**: Projector projecting horizontal gray-codes. **Right**: Projector projecting vertical Gray Codes.

To achieve this calibration, we capture images of a checkerboard where one of the squares has been partially filled with black to enable unique identification of each individual checkerboard corner (Figure 6). The calibration was performed in freshwater; for realistic usage scenarios, the calibration would need to be performed with the appropriate salinity level of the application site to ensure the correct refraction index of the water. To enable the simultaneous calibration of the projector, we project a series of Gray Code and line images onto the checkerboard (both horizontal and vertical) that allows us to uniquely resolve which projector pixel illuminated every object patch observed by the individual camera pixels. While other more advanced methods exist for unique identification (e.g., ChAruco boards [23]), our approach has the benefit of being more tolerant towards turbidity.

We first find the checkerboard locations using the checkerboard detector of Matlab r2024a, and we subsequently detect the black-filled square that allows us to uniquely identify the corners $X\left(a,b\right)$ across the two cameras for the integers a,b that span the checkerboard size, and where a,b indicate horizontal/vertical corner location relative to the top-left of the black corner. For the two cameras, we now have the camera pixel location $\left(x_{C,a},y_{C,b}\right)$ for each camera C∈[1,2] and corner. By decoding the projected Gray Code signals, we can determine the projector pixel $\left(x_{p,a},y_{p,b}\right)$ illuminating each of the corners $X\left(a,b\right)$.

We can now proceed to use Zhang’s camera calibration method [24] to estimate both the camera and projector intrinsics. The camera intrinsics can be estimated directly from the checkerboard locations $\left(x_{c,a},y_{c,b}\right)$ in the camera images, the projector intrinsics can be found similarly by using the coordinates $\left(x_{p,a},y_{p,b}\right).$ While dedicated calibration methods exist for subsea calibration [25], we have instead optimized our optical path by minimizing the distance between the lens and the flat port, reducing the impact of the water–glass interface sufficiently such that radial/tangential distortion parameters are sufficient to calibrate the system effectively.

Using the unique corner identifications, we can then also perform a stereo calibration between the three optical systems: Camera 1–Camera 2, Camera 1–Projector, and Camera 2–Projector. This calculates the relative rotation and translation of the three systems relative to each other, and allows us to use both pairs for 3D reconstruction, and also to convert 3D data found in one pair into the coordinate system of another pair. We furthermore project the two camera images onto a virtual common image plane to further speed up some of the calculations [26,27].

### 3.2. Scatter Removal Through a Dual Camera and Projector Setup

Based on discussions in Section 2, scatter will influence the quality of 3D measurements in turbid media. When probing the scene with a thin line, a spatiotemporal high-pass filter will robustly remove (most of) the backscatter envelope, as this envelope is slowly varying in both time and space. However, the noise on the backscatter envelope (shot noise, marine snow, etc.) is not easily removed. Similarly, forward scatter carries with it the spatial frequencies of reflectivity in the scene, meaning that it can exhibit high-frequency variations in time and space when scanning the scene. In such a complex landscape, where the relative signal intensities and spatiotemporal characteristics can vary significantly, any filter will require significant a priori knowledge of the scene and water characteristics, and it will, in any case, be difficult to guarantee the quality of the filtering process [17]. Thresholding on intensity will inevitably also filter out physical but dark objects.

**Figure 7:**
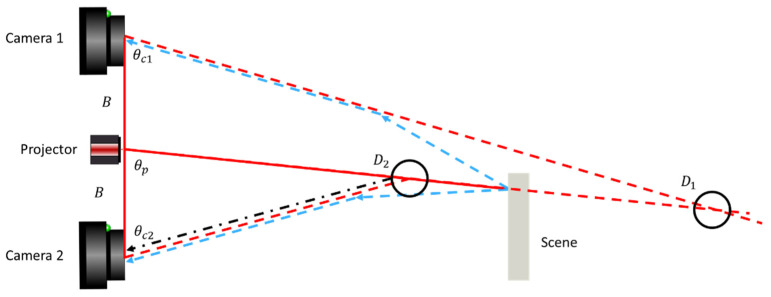
Dual-camera setup with a projector for scatter identification and removal. The projector emits a single line onto the scene, received by Camera 1 and Camera 2. Direct transport (solid red lines) can be distinguished from the forward-scatter transport (dashed blue lines) as the triangulation points $D_{2},\;D_{1,2}$ differ for the forward-scatter transport.

To overcome these limitations, we propose a dual camera and projector setup for identification and removal of false 3D points from scatter, shown schematically in Figure 7. Two cameras are placed at an equal distance from a central projector. The projector emits a single line perpendicular to the baseline of the three system components onto the scene, as described in Section 3.1. Lines are chosen as these patterns have previously been shown to be most robust against turbid waters [20]. Furthermore, unlike sine-based patterns, line-based patterns allow multiple candidates to be resolved, as illustrated in Figure 3. This provides additional robustness towards both forward scatter and backscatter. Like the situation shown in Figure 4, we image outside of an object. Again, a forward-scatter signal peak is observed once the projected line at $\theta_{p}$ hits an object in the scene. This peak will through triangulation at $\theta_{c1}$ and $\theta_{c2}$ be interpreted as an object at positions $D_{1}$ and $D_{2}$. The mirror symmetry of the Camera 1–Projector and Camera 2–Projector sub-systems will cause these positions to be inconsistent in the case of forward scattering, whereas true 3D observations will remain consistent.

**Figure 8:**
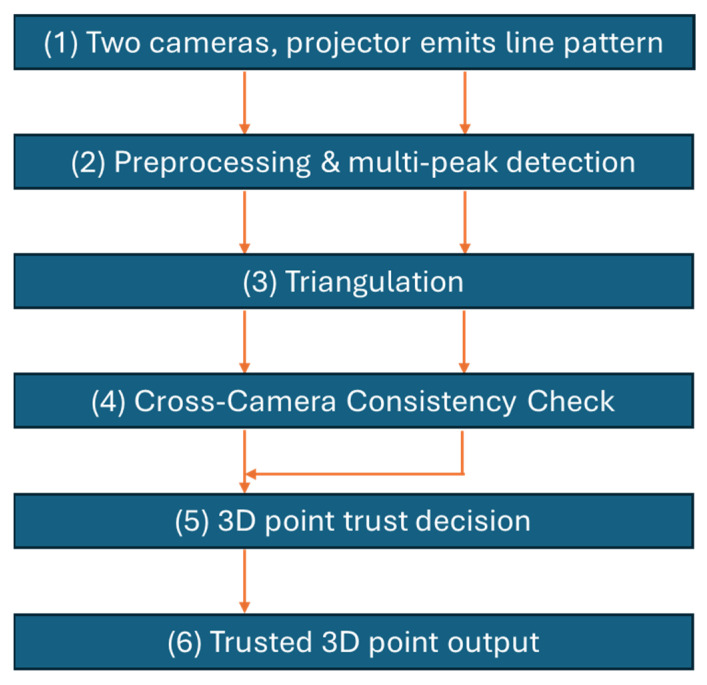
Overall flow chart for the algorithm. Steps 1–3 are performed per camera using a regular stereo/camera–projector 3D triangulation pipeline (dual vertical arrows). Steps 4 and 5 (this paper) introduce the cross-camera consistency check that enables trust classification and forward-scatter removal.

Our claim is that we can use the inconsistency in 3D data from scatter to filter out these false data points, allowing us to establish a trust classification scheme that will enable filtering of data points from scatter while keeping the detection limit as low as possible. We do this by cross-checking 3D points across the two cameras, taking advantage of the fact that forward scattering appears differently in the two cameras. Figure 8 shows a flowchart for the overall algorithm.

The 3D reconstruction process starts by first finding candidate 3D points in each of the camera-projector pairs. In practice, such candidate points are detected in the signal through a peak detection algorithm. One may choose to select a multitude of candidate peaks to increase the probability that the correct signal peak is within the selected set, but at the increased risk that false detections occur by chance. We choose to perform spatiotemporal high-pass filtering of the patterns to perform a first reduction in backscatter and forward scattering, similar to the method previously employed for time-of-flight imagery to reduce scattering [21]. As argued in Section 2, the true signal peak will also retain its spatial frequency in the presence of scatter, meaning that a filter can be designed that does not significantly reduce the signal strength even across a wide range of turbidities. This pre-filtering is efficient at removing the broad backscatter envelope, as argued in Section 2.2. Forward scatter, shot noise, and marine snow are examples of events that may contain high spatiotemporal frequencies, as argued in Section 2.2, where high-pass filtering alone cannot be guaranteed to isolate the true signal peak. However, as a first filtering stage, a high-pass filter reduces the number of candidate peaks required for subsequent processing, thereby simplifying processing and making the method more robust.

All the images are stacked into a 3D cube $I_{c}(m,n,p)$ where m,n is the row/column of the images, p is the pattern index (and represents time), and C∈{1,2} represents camera index. We subsequently detect two peaks per camera pixel in both of the cameras, i.e., find the two strongest peaks $P_{c}\left(m,n,i\right)$ in the p dimension of each of the $I_{c}$ cubes. Peaks are determined by finding local maxima and subsequently measuring their amplitude relative to a zero signal, selecting the two strongest peaks using amplitude as a metric. Peaks weaker than a fixed threshold are discarded. The peaks represent practice columns in the projector, and we use the camera–projector calibration to perform triangulation between the camera pixel and the projector column. This provides up to two 3D coordinates $Z_{c}\left(m,n,i\right)$ per camera pixel in each of the cameras.

We now want to use independent observations to verify whether results are consistent across cameras. As described above, real observations should provide the same results across cameras, whereas scatter observations should provide different results across cameras. We therefore proceed as follows for each 3D camera measurement $Z_{1}\left(m_{1},n_{1},i_{1}\right)$ in Camera 1:

1. Using the camera calibration between Camera 1 and Camera 2, determine the pixel $\left(m_{2}^{\prime },n_{2}^{\prime }\right)$ in Camera 2, where $Z_{1}(m_{1},n_{1},i_{1})$ should have been observed. As the camera is calibrated, this is simply a rotation/translation of the 3D coordinates $Z_{1}\left(m_{1},n_{1},i_{1}\right)$ into the coordinate system of Camera 2, yielding $Z_{1}^{\prime }\left(m_{1},\;n_{1},i_{1}\right)$ followed by a homogeneous transform of the 3D coordinates $Z_{1}^{\prime }$ into the 2D image plane ${(m}_{2}^{\prime },n_{2}^{\prime })\;$ of Camera 2.
2. For each $Z_{1}^{\prime }\left(m_{1},\;n_{1},i_{1}\right)$ of the transformed measurements from Camera 1, find the 3D camera measurement $Z_{2}\left(m_{2}^{\prime },n_{2}^{\prime },i_{2}\right),\;i_{2}\in \{1,2\}$ that is closest in Camera 2 as described in point 1. As the pixel indices $\left(m_{2}^{\prime },n_{2}^{\prime }\right)\;$ in Camera 2 for searching the closest measurements have been found through calibration, this process can be performed very quickly without the need for, e.g., kd-trees [28].

By operating in XYZ space instead of projector-column space, we can reduce the mounting accuracy requirements on the actual camera rig, as we do not need, e.g., all the optical centers to be on a line. Furthermore, all operations are constant-time, making the algorithm executable in O(1) and thus suitable for real-time implementations.

Due to shadowing and low signal-to-noise ratios, not all points in the scene will be observed by both cameras. This means that the camera pixels may contain zero, one, or two 3D measurements. Furthermore, this means that the procedure above will end up in one of the following three distance classes for each $Z_{1}^{\prime }\left(m_{1},n_{1},i_{1}\right)$:

1. A point $Z_{2}$ is found close to $Z_{1}^{\prime }$ in Camera 2, e.g., ${\left|\left|Z_{2}-Z_{1}^{\prime }\right|\right|}_{2}<t$ where t is a suitable threshold. (“Close”)
2. A point $Z_{2}$ is found far away from $Z_{1}$, e.g., ${\left|\left|Z_{2}-Z_{1}^{\prime }\right|\right|}_{2}\geq t$ where t is a suitable threshold. (“Far”)
3. The pixel $Z_{2}(m_{2}^{\prime },n_{2}^{\prime })$ does not contain any 3D measurements (no peaks above threshold), meaning no match can be found (“None”).

The threshold *t* is selected to reflect the overall system inaccuracy, and will relate to system parameters like the selected baseline, distance to objects, camera/projector resolution, and signal-to-noise ratio [29], meaning that it will need to be tuned to the hardware at hand.

Our key observation is that for a non-scattered, true 3D object measurement, a maximum of one 3D data measurement in Camera 1 should have a maximum of one 3D data measurement in Camera 2 that is close. This is based on the following considerations:

- We only have opaque surfaces that are to be imaged. This means that there will only be one true 3D measurement per camera pixel for either camera. If we detect a secondary peak, that is due to scattering.
- Based on the consideration illustrated in Figure 7, we see that scattered data points will differ in 3D location between the two cameras, meaning that these are highly probable not to be “close”. The correct measurements will, however, always align closely with each other.

More formally, the 3D observations in each camera pixel for the camera C will be in the set $O_{C}(i)=\left\{\{\emptyset ,\emptyset \},\left\{S_{C},\emptyset \right\},\left\{T_{C},\emptyset \right\},\left\{T_{C},S_{C}\right\},\left\{S_{c},S_{C}^{\prime }\right\}\right\},C\in [1,2]$, where $T_{C}$ refers to a 3D measurement from a true surface, $S_{C},S_{C}\prime$ are measurements due to scattering, and i∈[1,2] is the measurement index. As the points have been transformed into the same coordinate system using the camera–camera calibration, we can reasonably assume that $\left|\left|T_{1}-T_{2}\right|\right|<t$ for true measurements. If one of the measurements $O_{C}\left(i\right)\in \left\{S_{C},S_{C}^{\prime }\right\}$ are due to scatter, then it is highly probable that $\left|\left|O_{C}\left(i\right)-O_{C}(i^{\prime })\right|\right|\geq t$. Furthermore, we would expect $\underset{i_{1},\;i_{2}}{\min}\left|\left|O_{1}\left(i_{1}\right)-O_{2}\left(i_{2}\right)\right|\right|<t$ only to be the case if $T_{C}$ is present in both sets.

Using this logic, we will report the 3D measurement that minimizes ${\left|\left|Z_{2}-Z_{1}^{\prime }\right|\right|}_{2}$ as the measurement for the pixel and only consider trusting the measurement if the measurement is classified as “Close”. We see from Table 1 that this most likely corresponds to the cases where both cameras have captured the true object measurement.

In practice, there will be cases where, due to noise fluctuations or other factors, scattered points randomly happen to correspond to true measurements, meaning that the logic needs to be made slightly more complicated to cover all relevant cases. Table 2 thus captures the full set of distance classes that the algorithm may receive as input, and the corresponding trust consideration of the resulting data. In particular, this table covers the cases where, due to random chance, both points are classified as being “close”—e.g., one true measurement where random noise causes the scattered point to also be aligned. The trust considerations in Table 2 can be summarized as follows:

- Trusted1 means that the assumption of the algorithm has been met—a single data point that can be verified between cameras.
- Trusted0 represents no data received, which can also be considered a safe state.
- Untrusted1 represents a single point that could not be verified. This will either be a scatter point or an object point that could not be verified in the second camera due to shadowing. Usually, this point will be disregarded as untrustworthy.
- Untrusted2 represents that both points reported have a nearby corresponding point. Typically, this occurs when the scattering is too close to the real object point to be well separated. In this case, no reliable selection can be made.
- Untrusted3 indicates that all points are far from each other. This typically happens if only scattering is measured by both cameras.

It should be noted that an alternative approach could be to reproject the coordinates into the projector (e.g., for each projector pixel, determining which camera pixels report this projector pixel as the most dominant). We note that it is more difficult to make an efficient algorithm for this approach, due to the nature of scattering, hundreds of camera pixels may observe the same projector column, whereas a single camera pixel will only observe a very low number of projector columns. In short, this makes our camera pixel-based algorithm approach bounded in terms of time and memory, whilst a projector pixel-based algorithm approach easily becomes unbounded due to the nature of scattering.

While we have seen that two peaks are usually sufficient (one for the real peak, second for the forward-scattering peak), one can easily extend the algorithm to, e.g., 3–4 peaks (again only trusting those cases where both cameras confirm detection of a point at the equivalent 3D locations), but this will increase the chances of false positives.

## 4. Results

**Figure 9:**
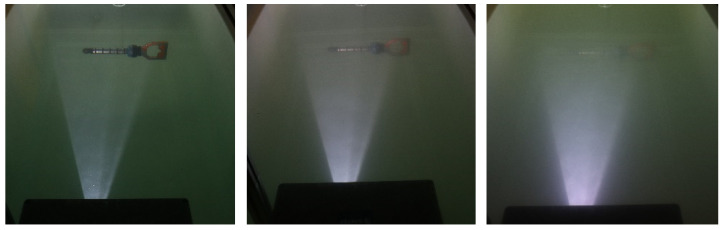
View of the imaging geometry for the camera at NTU 1.4 (**left**), NTU 3.3 (**center**), and NTU 6.0 (**right**) observing a hot stab.

**Figure 10:**
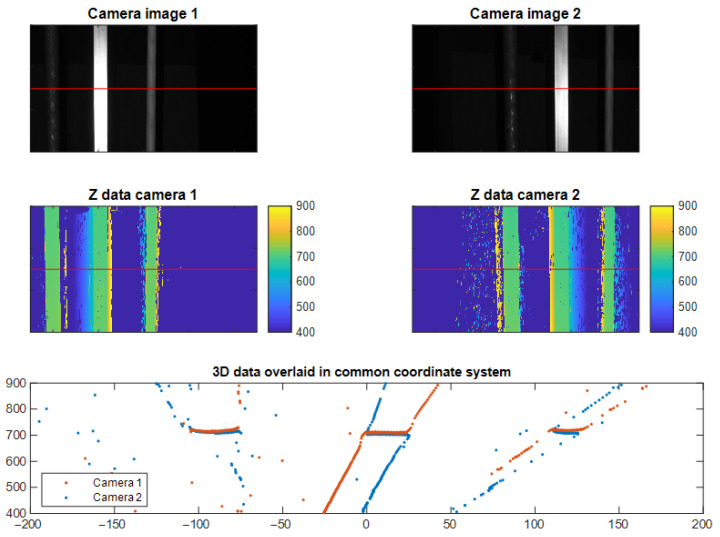
Example experimental data illustrating how scatter affects the two different viewpoints differently. The top row shows a camera image from both cameras after stereo rectification. The middle row shows the corresponding 3D data recovered from each camera view. The bottom row shows 3D data of a single row in the 3D data (indicated by a red line) from both cameras transformed into a common coordinate system.

The measurement geometry is shown schematically in Figure 7. The camera is placed in a white plastic tank, measuring 2 × 1 × 1 m^3^ (L × W × H),. We use clay as a scattering medium, following the procedure described in [20]. Using a turbidimeter to measure NTU, we repeatably measure a turbidity increase of approximately 0.4 NTU/(mg/L) of clay added to the water volume. Experiments are conducted at NTU 0.3–8.0. For a visual impression of NTU, sample images are found in Figure 9. For test purposes, we image a set of metal rods in front of a black surface (Figure 10, top row). The distance between the rods and the camera is 0.7 m. 

### 4.1. Qualitative Assessment

Figure 10 provides a qualitative display of the principle of our method. We see the two images from the two camera views, together with the recovered 3D data. The 3D data in either camera has been generated by performing triangulation between the projector and each camera. We see that scattering causes false 3D data to be generated in both views, but we also see that the relative position of the scattering is different for the two cameras.

To the left of objects, the false data points due to scattering manifest as being closer to the camera in Camera 1, whereas they manifest as being further away for Camera 2. The same difference in distance reported for scattered data points is on the right side of the objects. This is in line with our hypothesis illustrated in Figure 7 and indicates the feasibility of our method.

**Figure 11:**
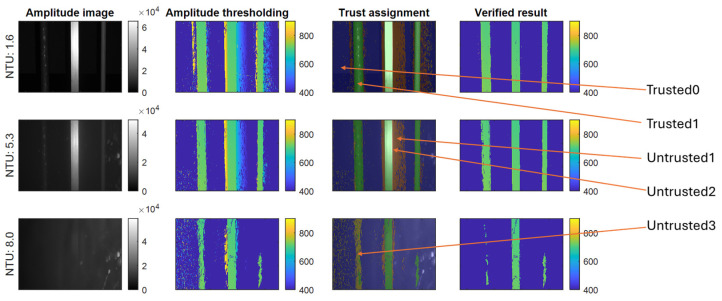
Example results from using the scatter recognition method, and results with degrading water qualities. Each row shows different turbidities. First column: Amplitude image. Second column: 3D data available after amplitude thresholding. Third column: Measurement classification result (see arrows also): Green/blue: Trusted1/trusted0 points (not scatter). Brown: Untrusted1 (not verifiable). Dark red: Untrusted2 (multiple points close). Beige: Untrusted3. Fourth column: 3D data after removing untrusted data points. Refer to Section 3.2 for an explanation of trust classes. Distances are reported in mm.

Figure 11 shows how the scattering is present in all of the turbidities, and qualitatively how our method detects it and removes it. We observe that the performance of the system is largely unaffected by turbidity until we reach a turbidity level where the signal-to-noise ratio of the objects is too low so that they can be reliably detected in both cameras. In this case, the system ends up reporting a very sparse set of 3D data.

### 4.2. Quantitative Assessment

**Figure 12:**
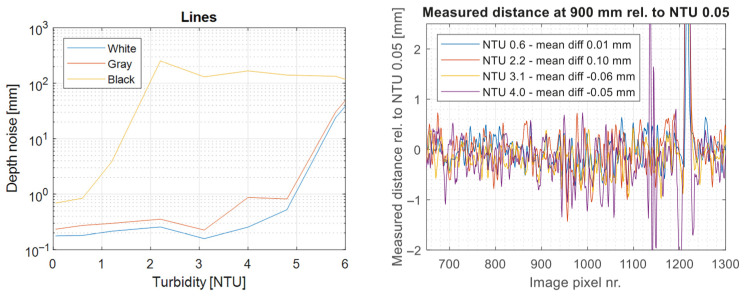
Left: Measured 3D precision vs. turbidity. Sub-mm precision on gray and white targets for NTU < 5. Right: Relative distance measured across a distance on a stationary target. Three-dimensional distance remains unaffected by turbidity within measurement error, i.e., less than 1 part in 10,000.

In this work, our performance goal is to be able to determine whether a 3D measurement represents a true 3D measurement, or whether it is a possibly false data point due to scattering or other imaging artifacts (shadows, etc.). This means that the usual 3D metrics like 3D precision and accuracy are not the primary focus of the work described herein and will not be directly relevant for providing useful performance values. That being said, it should be noted that the method described in Section 3.2 is purely a filtering method, and that a valid 3D point is a point that is measured by both camera systems. As such, a naïve implementation to assess 3D performance would be to use, e.g., all verified 3D data points as measured by Camera 1. In this implementation, 3D precision is equal to that reported in [20], i.e., 0.2 mm precision at 1 m distance for turbidities < NTU 4. For completeness, the measured 3D precision vs. turbidity is shown in Figure 12. Measured 3D at 900 mm distance varies by <0.1 mm across turbidities, meaning that the calibration is unaffected by turbidity to approximately 1 part in 10,000.

For this work, we consider the relevant metric to be that of a classification problem. The 3D camera system will report a series of measurements that either correspond to real 3D data or are artifacts of scattering and should be disregarded. The 3D camera system, therefore, needs to report both the 3D measurements and whether the 3D measurement is to be trusted or not. For many relevant applications, a simpler requirement can be formulated: the system should only report true 3D measurements, and as many true 3D measurements as possible.

Based on this requirement, we therefore consider the problem a binary classification problem. A 3D scene can be decomposed into two categories: parts of the scene that provide real 3D data (positives), and parts of the scene that contain nothing/void (negatives). As a baseline, we consider a method solely based on amplitude thresholding and a distance range, selecting the strongest peak per camera pixel. We note that such a method will effectively need to carefully balance the removal of scattering against not removing dark objects. For our method, we only consider the measurements classified as Trusted1 as positives, and the remaining as negatives.

**Figure 13:**
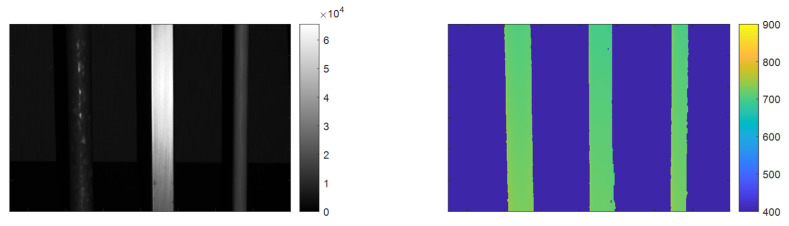
Experimental scene for quantifying algorithm performance. Data captured clear water and was processed to be used as ground truth. **Left**: Amplitude signal. **Right**: Corresponding 3D data. Depth in millimeters.

To quantify the performance, we use data at approximately 0 NTU (clear water) as our ground truth for determining whether a camera pixel should contain 3D data or not, shown in Figure 13.

We can further analyze the data quantitatively by considering the false positive and negative rates, FPR=FP/(TN+FP) and FNR=FN/(TP+FN), where TP,TN,FP,FN represent true/false positives and negatives. These metrics provide information about the proportion of the population where the test result is incorrect. These results are summarized in Table 3. As we can see, the method provides a 50–100× improvement in the false positive rate for all turbidities, whilst maintaining an almost zero false negative rate.

It is interesting to notice that the amplitude-based baseline method actually improves with increasing turbidities. This may be slightly surprising, but by referring to Figure 11, we see that this is purely due to the fact that overall fewer 3D points are reported. As the amplitude threshold is highly dependent on water turbidity, the attenuation of the water will result in fewer observations for higher turbidities, thus implicitly “improving” results.

## 5. Discussion

The proposed filtering and classification method requires high-quality co-calibration of the camera–projector–camera geometry. Figure 12 shows that the calibration remains consistent to within 1 part in 10,000 over the course of hours. For real-life implementations, mechanical and thermal stability are critical parameters for any 3D camera, including this. Deployment in seawater will require calibration in representative salinity, as salt increases the refractive index of water by ~1%.

As has been discussed in previous sections, spatiotemporal filtering often requires parametric fitting to the water quality in question. For our method, spatiotemporal filtering is simply a signal conditioning stage, and our results demonstrate that this filtering performs sufficiently across a range of turbidities without any fitting parameters. The method requires relatively good calibration between both camera–projector pairs and the camera–camera pair, but as seen in Figure 12, this can be achieved and maintained across turbidities.

It has previously been demonstrated that Time of Flight (TOF) systems can reliably filter out backscatter [19] through range-gating. However, forward scatter will not be removed through range-gating. The reason for this is straightforward: TOF systems measure the return delay of a light pulse, and backscatter can be filtered out simply because it arrives first at the sensor. Forward scatter carries only a very moderate added time delay (on the order of centimeters) and will, as such, be interpreted as an object at the distance of the scattering object, and cannot be removed in any straightforward manner, as it is easily interpreted as an extension of the scattering object. As such, the filtering of forward scatter requires other methods, such as a spatial unsharp filter [21], which, as a side effect, also removes the dark parts of the scene.

This problem, related to the geometry of forward scatter, also affects structured light systems, where forward-scattered light again leads to poorly defined object boundaries. In this paper, we propose a measurement geometry, pattern set, and algorithm that ensures that detections caused by forward scatter produce inconsistent 3D measurements, allowing them to be identified and filtered out. For TOF systems, such an “inverting” geometry is not straightforward to produce. Adding a second TOF camera will simply produce a common-mode error that cannot be filtered.

## 6. Conclusions and Summary

We have presented a first-principles method for eliminating false data points due to scattering for subsea imaging. The method is based on the combination of a camera–projector–camera geometric setup and projected line patterns that ensure that false data points due to scattering can easily be recognized and thus removed from the final 3D data. The implementation is performed in a mixture of XYZ space and camera sensor space, making it both fast and tolerant for minor inaccuracies in camera–camera–projector alignment.

In experiments, we see a 50–100× reduction in the false positive rate across all water turbidities compared to the baseline algorithm, whilst maintaining a false negative rate close to zero. For real applications, this means that our method provides a clear indication of whether a data point is to be trusted as a real 3D measurement.

While other methods for scatter removal exist, primarily based on different forms of spatiotemporal image filtering, these methods often require fine-tuning to the particular water quality at hand, making them unreliable in real-world deployments. Furthermore, they do not provide a trust classification of the 3D data, which can be prohibitive for robotic applications. By contrast, our method upholds performance without parameter tuning even for drastic changes in water turbidity. That being said, our method can be combined with pre-filtering, which is indeed performed in the work presented here.

The hardware and method presented produce 3D point clouds with megapixel-level Xy resolution, sub-millimeter precision, and sharp, well-defined object boundaries. This makes the approach highly suitable for subsea interaction tasks such as inspection, maintenance, and repair, as well as intervention using Remotely Operated Vehicles (ROVs). However, its most compelling application may be in reliable free-space detection for collision avoidance, both for conventional ROVs and for autonomous systems, where existing methods often struggle to distinguish dark objects from false returns caused by forward scatter.

In summary, our method provides a novel, turbidity-independent method for managing scattering in water, paving the way for even more robust 3D imaging systems in the future.

## Figures and Tables

**Table 1:** Confusion table for the different cases of 3D measurements from the two cameras. Expected classification of measurements based on the properties of scattering.

$O_{2}\left(i\right)$ $\backslash O_{1}\left(i\right)$	∅	$\left\{S_{1},\emptyset \right\}$	$\left\{T_{1},\emptyset \right\}$	$\left\{T_{1},S_{1}\right\}$	$\left\{S_{1},S_{1}^{\prime }\right\}$
∅	None	None	None	None	None
$\left\{S_{2},\emptyset \right\}$	None	Far	Far	Far	Far
$\left\{T_{2},\emptyset \right\}$	None	Far	Close	Close	Far
$\left\{T_{2},S_{2}\right\}$	None	Far	Close	Close	Far
$\left\{S_{2},S_{2}^{\prime }\right\}$	None	Far	Far	Far	Far

**Table 2:** Confusion table for the classification of the two different points, and the corresponding decision on whether the measurement in a camera pixel can be trusted. Points 1 and 2 reflect the resulting classification according to Table 1 of the two per-pixel peaks in the camera.

	Point 1 Classification		
Point 2 Classification	None	Close	Far
None	Trusted0	Trusted1	Untrusted1
Close	Trusted1	Untrusted2	Trusted2
Far	Untrusted1	Trusted2	Untrusted3

**Table 3:** False positive and negative rates for our method and baseline amplitude method as a function of turbidity (NTU). All numbers are given as percentages.

	False Positive Rate (%)		False Negative Rate (%)	
NTU	Our	Amplitude	Our	Amplitude
0.3	0.7	31.0	0.0	0.0
1.6	0.5	29.6	0.1	0.0
2.4	0.3	25.6	0.1	0.0
3.5	0.3	22.2	0.1	0.0
4.5	0.1	17.2	0.1	0.0
5.3	0.1	17.4	0.1	0.0
6.5	0.0	10.5	0.4	0.2
8.0	0.0	7.6	0.6	0.4

## Data Availability

Data underlying the results presented in this paper are not publicly available at this time but may be obtained from the authors upon reasonable request.
